# Deubiquitinases in Ovarian Cancer: Role in Drug Resistance and Tumor Aggressiveness

**DOI:** 10.7150/ijbs.100355

**Published:** 2024-09-23

**Authors:** Giovanni Luca Beretta, Matteo Costantino, Luca Mirra, Pietro Pettinari, Paola Perego

**Affiliations:** Molecular Pharmacology Unit, Department of Experimental Oncology, Fondazione IRCCS Istituto Nazionale dei Tumori, Via Amadeo 42, 20133 Milan, Italy.

**Keywords:** deubiquitinases, ovarian cancer, drug resistance, proteasome, deubiquitinase targeting

## Abstract

Ovarian cancer is a lethal disease due to late diagnosis and occurrence of drug resistance that limits the efficacy of platinum-based therapy. Drug resistance mechanisms include both tumor intrinsic and tumor microenvironment-related factors. A role for deubiquitinases (DUBs) is starting to emerge in ovarian cancer. DUBs are a large family of enzymes that remove ubiquitin from target proteins and participate in processes affecting drug resistance such as DNA damage repair and apoptosis. Besides, DUBs modulate the functions of T cell populations favoring an immune suppressed microenvironment. Three DUBs are proteasome-associated, whereas the large majority are not. Among the former DUBs, USP14 has been proposed to modulate transcription factors such as Bcl6 and BACH1. In addition, RPN11/PSMD14 interferes with various processes including epithelial mesenchymal transition, also favored by non-proteasomal DUBs such as USP1 by acting on Snail. Besides, USP8 by stabilizing HER family receptors can confer drug resistance. Overall, DUBs appear to be druggable, with several inhibitors under development. Based on DUBs biological role, DUBs targeting appears promising in view of combination strategies involving different therapeutic approaches. Here, we summarize the relevance of DUBs in ovarian carcinoma and provide insights into future challenges for the treatment of this disease.

## Introduction

Ovarian carcinoma is not a frequent cancer, but it is often lethal because of its late diagnosis and the occurrence of drug resistance 1,2. The armamentarium of drugs available for ovarian carcinoma treatment is quite poor, although it has been recently enriched by the approval of inhibitors of Poly-ADP ribose polymerase (PARP) both for BRCA1 and BCRA2 mutant cancers as well as for the maintenance therapy of non-mutant platinum-sensitive recurrent ovarian cancer 3. The first-line treatment of ovarian carcinoma is a platinum-based regimen involving the combination of cisplatin or carboplatin with paclitaxel. In spite of the efficacy of the combination, drug resistance can develop 2.

The mechanisms of resistance to platinum agents have been deeply addressed mainly in preclinical models, representative of different tumor types, and they have been linked to multiple alterations affecting drug accumulation/efflux, drug sequestration by thiols, cell response to DNA damage and DNA repair 4. In ovarian carcinoma, a major contribution of cell intrinsic factors to drug resistance has been clearly defined, with the recognition of a major role of impaired apoptotic response due to mutation of TP53, already in early studies 5. The main DNA repair alteration underlying resistance to platinum agents is an increased expression of enzymes of the Nucleotide Excision Repair pathway, with ERCC1 being proposed as a biomarker of response to platinum-based therapy 6. An additional mechanism of resistance relevant in ovarian carcinoma includes restoration of homologous recombination (HR) in tumor cells originally bearing mutations in the BRCA1 or BRCA2 genes. The available evidence also supports the relevance of pathways sustaining cell survival, such as the PI3K-Akt-mTOR and RAS-RAF-MEK pathways in conferring resistance to platinum agents 7,8. Drug resistance of ovarian carcinoma is not simply related to tumor intrinsic features, but a role for the tumor microenvironment has been documented 9. For instance, cancer-associated fibroblasts (CAFs) have been reported to support survival of tumor cells exposed to cisplatin due to the production of thiols (glutathione and cystine), that can - however - be disabled by the presence of γIFN producing T cells 10. Such cells by up-regulating γ-glutamyltransferases and transcriptional repressing system xc (-) cystine and glutamate antiporter via the JAK/STAT1 pathway prevent production of thiols by CAFs and therefore tumor drug resistance.

A role for DUBs in supporting the growth of tumor cells resistant to platinum agents emerges from the literature. DUBs are enzymes that act by catalyzing the detachment of ubiquitin from protein substrates. They represent a large family of proteins with around 100 members, mainly belonging to two subfamilies, i.e., the ubiquitin-specific peptidases/proteases and the Zn-dependent ones as well as pseudo-enzymes devoid of enzymatic function. The key role of DUBs in cancer has been recently reviewed by Dewson and colleagues who described their contribution to cancer survival with specific reference to the hallmarks of cancer 11. Such hallmarks have increased with time, but already in 2000 a major contribution of signaling sustaining tumor cell survival was recognized 12. Indeed, regarding the interference of DUBs with cancer hallmarks, a layer of complexity is provided by the modulation of tumor cell survival pathways by DUBs. For example, a regulation of the stability of receptors of the HER family by Ubiquitin-Specific Protease 8 (USP8) has been documented 13-15. This phenomenon is relevant given the role of such receptors as therapeutic targets as well as their involvement in growth factor and PI3K signaling. The available evidence also suggests that DUBs act in the tumor microenvironment because they can play a role in modulating the functions of various T cell populations. In this regard, USP8 may lead to cancer immune evasion as it supports regulatory T cells and suppresses T cell functions 16. In this regard, it has to be noted that ovarian cancer resides in an immunosuppressed microenvironment that favours progression and drug resistance 17.

Based on this background, here we review the role of DUBs in drug resistance of ovarian carcinoma with specific reference to the exploitation of their targeting in this disease.

### The deubiquitinase family: classification and functions

**Figure 1:**
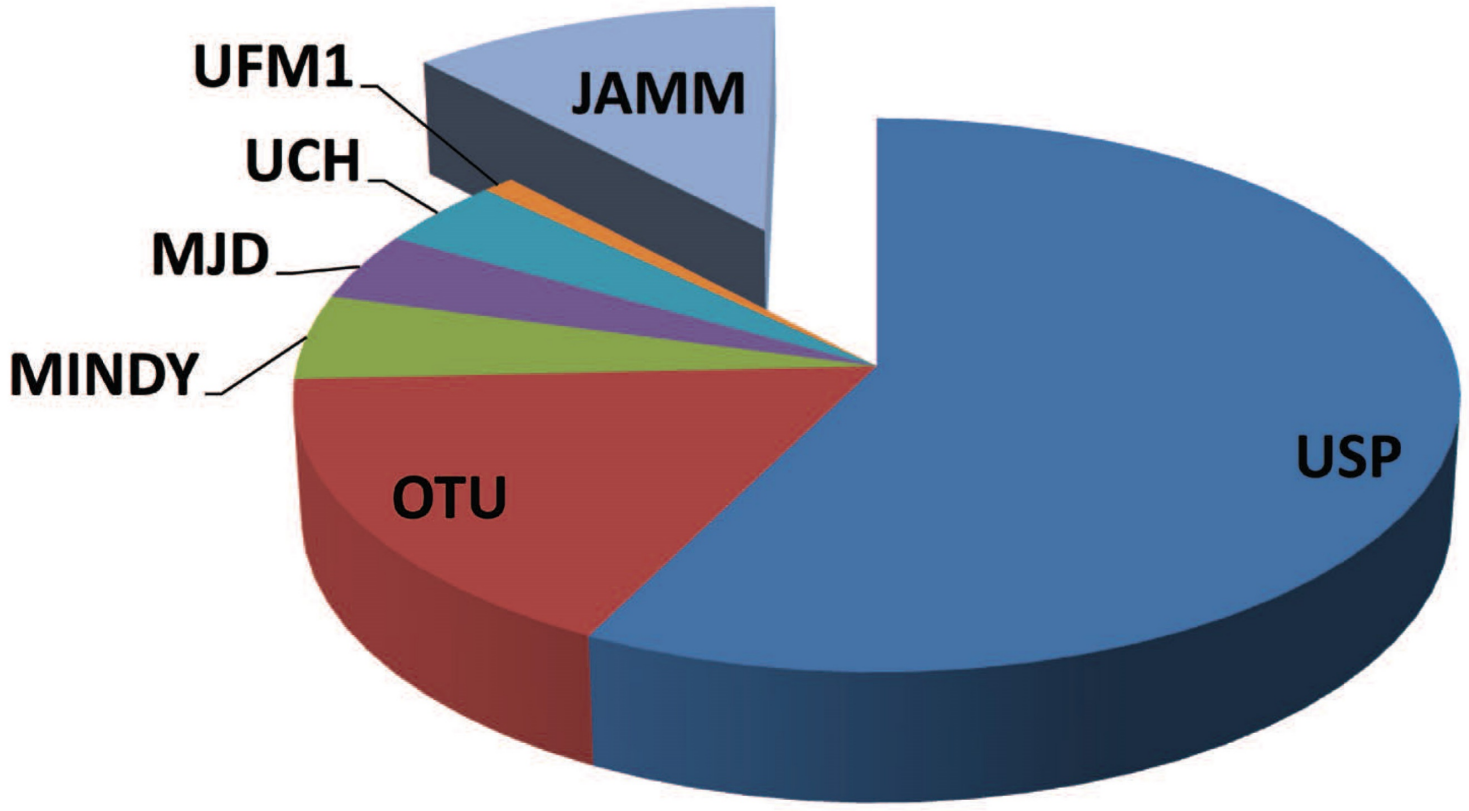
**The family of deubiquitinases.** A graphical representation of the different groups of deubiquitinases showing the heterogeneity of the components and their relative distribution across the family. Cysteine proteases: UCH, ubiquitin C-terminal hydrolase; USP, ubiquitin-specific protease; OTU, ovarian tumor domain-containing proteases; MJD, Machado-Josephin domain-containing proteases; MINDY, K48 polyubiquitin-specific MIU-containing novel DUB family domain-containing proteases; UFM1, Ubiquitin Fold Modifier1-specific peptidase domain-containing proteases. Metallo proteases: JAMM, JAB1/MPN/MOV34 domain-containing enzymes.

DUB enzymes can be grouped in two major classes: cysteine proteases (CP) and metallo proteases (MP) (Figure 1) 18,19.

In humans, the CP class is the best characterized one and includes over 80 proteins. These enzymes have been mechanistically and structurally studied as well as proficiently inhibited 20-22. Based on their aminoacid sequence and protein domain organization, CP are classified in six families including: the ubiquitin C-terminal hydrolases (UCHs), the ubiquitin-specific proteases (USPs), the ovarian tumor domain-containing proteases (OTUs), the Machado-Josephin domain-containing proteases (MJD), the K48 polyubiquitin-specific MIU-containing the novel DUB family domain-containing proteases (MINDYs), and the zinc finger with Ubiquitin Fold Modifier1-specific peptidase domain-containing proteases (UFM1s) 23. To hydrolyze the ubiquitin attached to the substrate, the active site of CP takes advantage of a cysteine, a histidine and often an asparagine or aspartate residue 19, 23, 24. The active or inactive condition of this type of DUBs relies on the presence of a reactive thiolate (-S^-^) or inactive thiol (-SH) group of the cysteine 25. Substrate binding, interaction with a protein complex as well as post-translation modifications of the DUB itself usually regulate conformational shifts and the state transition 26-29. The conformational changes polarize the active site histidine, commonly mediated by the presence of an asparagine or aspartate residue 30,31. Following the histidine polarization, the pKa of the cysteine is lowered and this feature induces thiol deprotonation and thiolate stabilization 26, 24. Thiolate cysteine undergoes a nucleophilic attack by ubiquitin/polyubiquitin (Poly-Ub) chain linked to the substrate, leading to the formation of a thioester intermediate. When the bond of the ubiquitin-DUB intermediate is hydrolyzed, substrate release occurs, and the CP DUB returns back to a thiolate reactivated DUB for a new enzymatic cycle 26,32.

The UCH family comprises four enzymes, i.e., UCHL1, UCHL3, UCHL5, and BAP1. UCHL3 is the first structurally characterized DUB 20. Peculiar of this family is the loop structure hiding the entrance of the active site that selects the substrates according to the size 20, 33. However, too large substrates can bypass this loop-mediated obstacle via their unfolding 33. Among UCH DUBs, BAP1 plays a critical role in controlling the cell-cycle and DNA damage response (DDR) 34. Mutations of BAP1 impacting on enzymatic activity and affecting germline cells predispose to melanoma and renal cell carcinoma 35 and are frequent in malignant pleural mesothelioma 36.

The USP family includes 58 members and is the largest one. Structurally, these enzymes appear as a hand with three major domains, i.e., a thumb, a palm, and fingers 27, 37, 38. The fingers stabilize the interaction with distal ubiquitin on substrates, while the catalytic residues are positioned between the thumb and the palm 27.

The OTU family, originally identified in *Drosophila melanogaster* 39, includes 16 members showing different specificity for ubiquitin substrates 40. The OTUs contain an OTU catalytic domain, an ubiquitin interaction domain (i.e., ubiquitin interacting motif), an ubiquitin associated domain, or Zinc finger domain 39. The different OTUs display specificity for different chains. In fact, specific for K48 chains is the ovarian tumor deubiquitinase, ubiquitin aldehyde binding (OTUB1). Specificity for K11 chains is reported for Cezanne, while ovarian tumor deubiquitinase (OTUD2) specifically acts on K11, K27, and K33 chains 40-42. The different substrate specificity implies that OTU DUBs regulate different cellular signalling pathways 40. Some OTU DUBs lack the asparagine/aspartate residue in the catalytic site 30. The absence of the negatively charged residue in the catalytic triad, which is essential to polarize the histidine, results in inactive DUB members, such as in the case of OTUB2. However, this behaviour is controversial following the observation that the Tumor Necrosis Factor alpha-induced protein 3 (A20) retains activity following an induced mutation of its catalytic aspartate residue 43, 44.

The MJD family contains four members, including ataxin 3 (ATXN3), ATXN3L, Josephin domain-containing 1 and Josephin domain-containing 2. Together with the catalytic cysteine, the highly conserved catalytic Josephin domain contains two ubiquitin binding sites and two histidines 23,45. The acronym MJD, meaning Machado-Josephin disease, reflects a neurological disorder produced by an amplification of the CAG sequence in ATXN3 leading to protein misfolding and aggregation 46.

The five members (MINDY1-4 and MINDY4B) of the MINDY family are specific for K48 ubiquitin linkages 23, 47. These DUBs, which are inactive prior to substrate binding, are activated by the interaction with their substrates that induce conformational changes required for enzyme activation 48.

The UFM1 family includes only one member. This enzyme contains two ubiquitin-binding domains, which are necessary for catalyzing the cleavage of K63 linkages 49.

The class of MP DUBs includes the zinc-dependent enzymes equipped with JAB1/MPN/MOV34 (JAMM) domain 18, 50. Among the 14 genes of the human genome containing this domain, seven coordinate the zinc ion cofactor, and only six of these are capable to hydrolyze ubiquitin conjugates, including AMSH, AMSH-LP, BRCA1/BRCA2-containing complex subunit 3, COP9 signalosome complex subunit 5, Myb-like, SWIRM and MPN domains 1, and regulatory particle non-ATPase 11 (RPN11) 51, 30, 52.

One aspartate, two histidine residues and one serine are contained into the catalytic site of the MP DUBs 18, 51. By using the zinc ion, MP hydrolyzes a water molecule producing the hydroxide ion allowing the hydrolysis of the isopeptide bond between substrate and ubiquitin 18. No covalent intermediate between the enzyme and substrate is formed.

**Figure 2:**
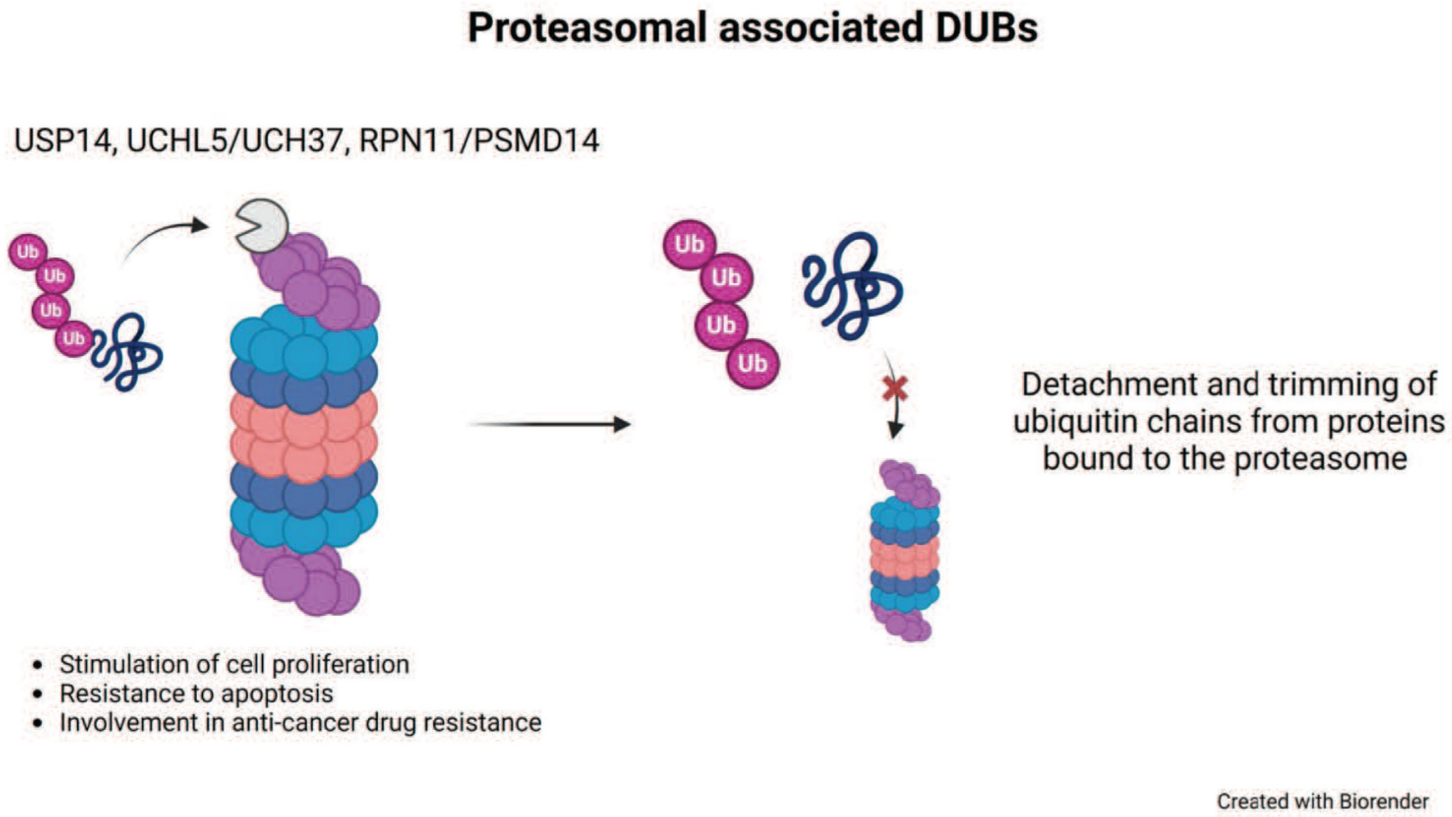
** Proteasomal deubiquitinases.** Representation of the small subset of proteasomal deubiquitinases that include three members and their major cellular functions. The complete proteasome structure consisting of a 20S core particle and two 19S regulatory particles is shown on the left. On top of the proteasome a schematic DUB displays the action on poly-ubiquitinated substrate proteins. Once the substrate is unfolded and ready for degradation, USP14 and UCHL5/UCH37 trim poly-Ub chains, whereas RPN11/PSMD14 realeases poly-Ub. USP, ubiquitin-specific protease; UCH, ubiquitin C-terminal hydrolase.

Among the MP DUBs, the best characterized and studied is RPN11/PSMD14. Together with two CP, USP14 and UCHL5, RPN11/PSMD14 associates with the proteasome (Figure 2) 53-55. The 26S proteasome complex contains a 20S catalytic barrel-shaped core, which interacts with different regulatory particles 56. By ubiquitin-binding receptors, the 19S regulatory particle recognizes ubiquitinated substrates with high specificity 57. Upon ubiquitin-binding receptor recognition, the poly-Ub chain is removed and recycled into the pool of free ubiquitin before substrate degradation 58. RPN11/PSMD14 acts on the link between the substrate and the proximal ubiquitin of the chain, thus the enzyme removes the complete poly-Ub chain at once 53, 58-60. Since RPN11/PSMD14 activity occurs upon ATPase dependent proteasome substrate unfolding, this feature delays its activity until the substrate is committed to proteasome degradation 59. The lack of a proficient RPN11/PSMD14 activity fails to remove the poly-Ub chain and this behaviour produces a steric hindrance of the substrate into the 20S core impeding protein degradation 59. Conversely, the regulatory functions of USP14 and UCHL5 trim the ubiquitin from substrates to limit their degradation 54, 61, 62.

AMSH is a MP DUB implicated in endosomal membrane trafficking. AMSH interacts with substrate proteins and removes K63 ubiquitin 63, 64. This peculiar behaviour of AMSH drives the fate of substrates allowing their degradation or recycling by the Endosomal Sorting Complexes Required for Transport (ESCRT) pathway 64. This feature renders AMSH an interesting target for therapeutic intervention 65.

### Deubiquitinases and drug resistance

Selected DUBs have been implicated in the molecular mechanism of ovarian cancer resistance (Table 1). For some of them, a clear role in the mechanism of resistance to clinically available agents has been established. Besides, a large body of evidence supports a contribution to features underlying tumor aggressiveness.

### Proteasomal deubiquitinases

#### USP14

Among proteasomal DUBs, USP14 is the one whose role has been better investigated following the discovery of the potential relevance of its targeting 66. In ovarian cancer, USP14 has been reported to be over-expressed as compared to normal tissue in early studies 67, 68. Of note, USP14 expression has been related to patient survival. A role for USP14 in regulation of cell proliferation and apoptosis has been reported in ovarian carcinoma cells with intrinsic resistance to cisplatin (i.e., SKOV3) in which the expression of USP14 has been shown to be cell-cycle dependent with an increase after serum addition in starved cells 68. USP14 has been shown to confer cisplatin resistance as its inhibition results in enhanced cisplatin cytotoxicity 69. In keeping with these studies, it has been reported that USP14 expression is augmented in cisplatin-resistant cells, where it confers resistance by stabilizing Bcl6 through prevention of its proteasomal degradation 70. Bcl6, a Zinc finger protein that acts as a transcriptional repressor, is known as an oncoprotein that supports proliferation as well as migratory and invasive abilities of ovarian cancer cells and behaves as a negative prognostic factor 71. Targeting of USP14 may therefore represent an alternative mode to inhibit Bcl6, besides the recently proposed direct targeting 72.

A recent report has highlighted a role for USP14 in the regulation of heme metabolism and of invasive cell abilities 73. In fact, by direct interaction, the DUB stabilizes the transcription factor BACH1 that acts by preventing the binding of Nrf2 to the promoter of HO-1, thereby decreasing HO-1 expression and reducing heme degradation. In addition, the activation of Nrf2 appears to promote USP14 expression, given that USP14 levels are correlated to Nrf2 levels across cancer types, oxidative stress and the disruption of the Keap1/Nrf2 interaction promote USP14 expression 73.

#### RPN11/PSMD14

RPN11/PSMD14 is a Zn^2+^ dependent protease belonging to the JAMM family that cleaves the ubiquitin chain allowing the substrates to proceed into the 20S core particle proteolytic chamber for degradation to be completed 74. A role for RPN11/PSMD14 in drug resistance is supported by multiple studies 75. A study of the molecular bases of aberrant autophagy in ovarian cancer which was found to be associated with an enhanced expression of the selective autophagy receptor SQSTM1/p62 has led to the identification of RPN11/PSMD14 as a negative regulator of autophagy levels 76. Through functional approaches in *in vitro* and *in vivo* models it has been demonstrated that RPN11/PSMD14 supports tumor aggressive features through a mechanism that involves Leucine Rich Pentatricopeptide Repeat Containing (LRPPRC).

Indeed, the deubiquitination following direct interaction results in inhibition of autophagy through LRPPRC/Beclin1-Bcl-2/SQSTM1 signaling pathway. Such observations are in keeping with the correlation between RPN11/PSMD14 and LRPPRC expression in clinical specimens 76. The C16orf72/HUWE1-associated protein modifying stress responses (HAPSTR1) has also been implicated in sustaining tumor progression in ovarian cancer through stimulation of epithelial mesenchymal transition (EMT) and inhibition of autophagy 77. Indeed, HAPSTR1 colocalizes with LRPPRC and inhibits its ubiquitination, with HAPSTR1 overexpression stabilizing LRPPRC through RPN11/PSMD14.

The available evidence supports that the contribution of RPN11/PSMD14 to ovarian cancer aggressiveness may derive from the cooperation among different mechanism, because it has been reported to decrease the enzymatic activity of pyruvate kinase M2 (PKM2). PKM2 represents a rate-limiting enzyme of glycolysis and is considered an important regulator of metabolic signals in cancer. PKM2 forms a tetramer and acts as a pyruvate kinase under normal conditions. Conversely, in cancer cells, PKM2 functions as a dimeric enzyme inducing the “Warburg effect”. RPN11/PSMD14 plays a role in regulating the post-translational modification of this enzyme. Specifically, RPN11/PSMD14 acts by reducing the ubiquitination on the K63 of PKM2 increasing the expression of the dimeric form and also favouring its nuclear translocation leading to aerobic glycolysis in tumors, including ovarian cancer 78.

### Non-proteasomal deubiquitinases

#### USP1

**Figure 3:**
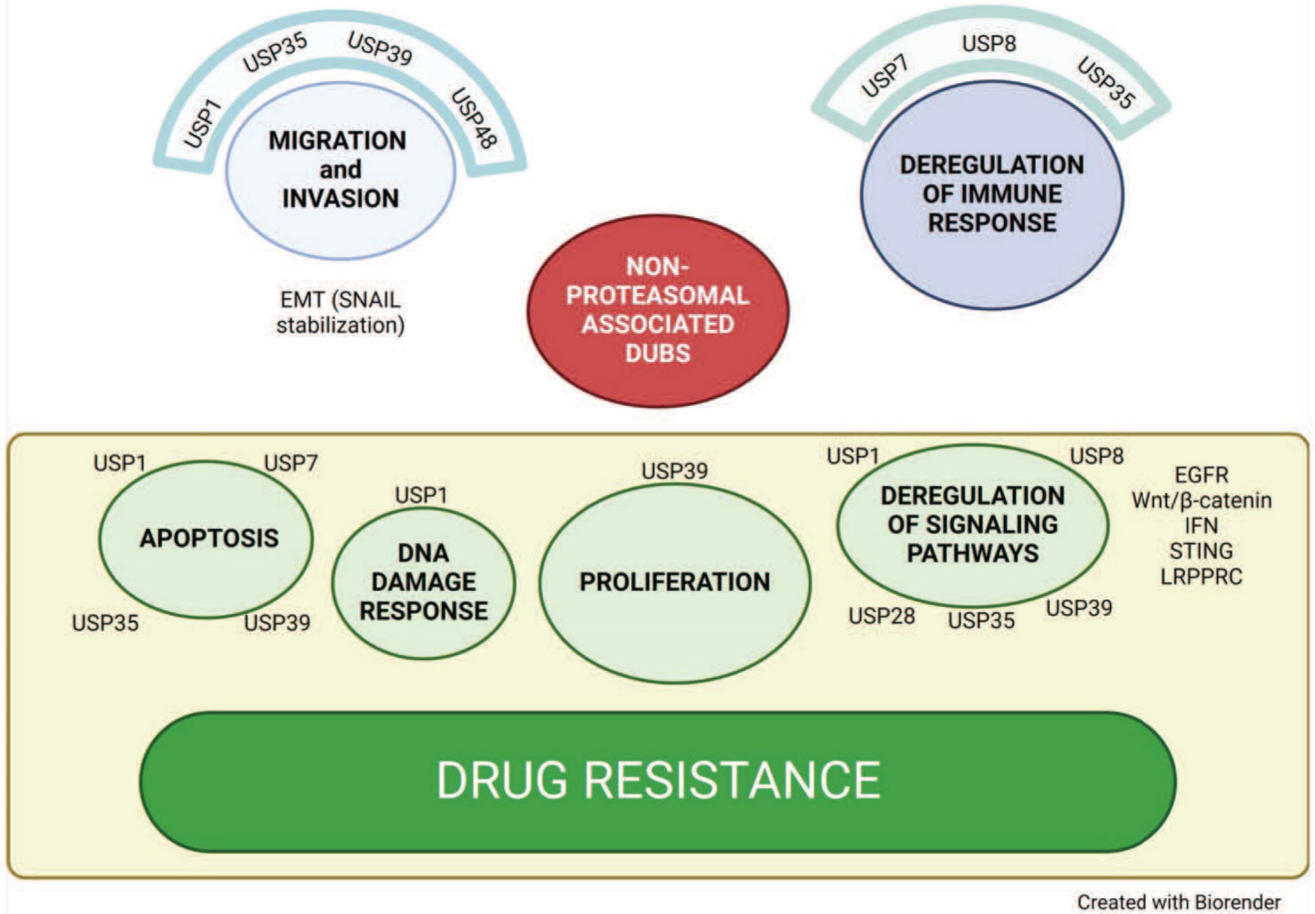
** Non proteasomal deubiquitinases.** Representation of biological functions of non-proteasomal deubiquitinases and their contribution to ovarian cancer aggressiveness and drug resistance.

USP1 plays a key role in the DDR, particularly in translesion synthesis of the Fanconi anemia (FA) pathways, by deubiquitinating PCNA and Fanconi anemia complementation group D2 (FANCD2), respectively 79, 30. USP1 contribution to the aggressive feature of ovarian cancer has been dissected out 80 and inhibitors of this DUB are being developed. USP1 has been implicated in conferring resistance to platinum agents and in favoring metastatic spread (Figure 3). Indeed, using a short-hairpin based loss-of-function screening for genes involved in apoptosis and in the DDR, USP1 was identified as a gene whose loss increases platinum sensitivity 80. Enhanced sensitivity to cisplatin was observed upon pharmacological inhibition of USP1 in keeping with an established role of USP1 in regulating the DDR by deubiquitinating FANCD2 81. Specifically, the DNA damage and genome repair is governed by the activity of the complex USP1-UAF1-RAD51AP1, which regulates FANCD2 ubiquitination/deubiquitination and in turn drug sensitivity 81. An additional function of USP1 is the regulation of EMT and stem cell state, in keeping with the evidence from the literature indicating that USP1 in complex with WD repeat domain 48 enhances TGF-β induced EMT of triple negative breast cancer cells via stabilizing TGF-β-activated kinase 1 (TAK1), also known as the mitogen-activated protein kinase 7 (MAP3K7) 82. In fact, silencing of USP1 resulted in decreased levels of genes associated with cancer stemness (e.g., Sox2, KLF4, c-Myc) under basal conditions and upon cisplatin exposure. In this context, a key observation is that USP1 regulates EMT by deubiquitinating Snail, thereby preventing its degradation. Physical interaction between USP1 and Snail has been documented to occur under basal conditions with an enhancement upon cisplatin exposure 80. A link between the DDR and EMT has been established, because cisplatin exposure of ovarian cancer cells results in USP1 phosphorylation by ATM and ATR, the two core kinases of the DDR; such a phosphorylation is required for binding to Snail, a process activating stemness feature that can ultimately induce cell reprogramming to favor survival.

USP1 dependency has been related to the aberrant processing of mono- and poly-Ub PCNA in BRCA1/2 mutant tumors as well as in BRCA1/2 wild-type cell lines including ovarian cancer cells 83. Following USP1 inhibition, a subset of BRCA1/2 wild-type as well as BRCA1/2 mutant cells show a S-phase specific DNA damage, decreased DNA synthesis, reduced replication fork speed, and activation of the ATR-Chk1 signaling cascade. RAD18 and UBE2K, which are responsible for mono- and poly-Ub of PCNA leading to reduced PCNA protein levels, mediate the observed USP1 dependency. Interestingly, the toxic PCNA protein degradation mediated by USP1 inhibition synergizes *in vitro* and *in vivo* with PARP inhibition in BRCA1/2 mutant tumors 83.

#### USP7

The DUB Herpesvirus associated Ubiquitin-Specific Protease/Ubiquitin-Specific Protease 7 (HAUSP/USP7) has been implicated in tumorigenesis with participation to several cancer hallmarks 84. USP7 plays a key role in the regulation of the p53/MDM2 axis 85 and *in vivo* knock out of the gene has been shown to result in increased p53, cell-cycle arrest and apoptosis. Enhancement of p53 levels as a consequence of knockout results in induction of BCC3/PUMA, CDKN1A/p21 and MDM2. Increased p53 is associated with enhanced degradation of MDM2 initially compensated by the MDM2 induction. USP7 seems to play a key role in different cancers, including ovarian cancer where it has been proposed as a prognostic factor 86.

The catalytic core domain of USP7 has been crystallized more than twenty years ago 87 and co-crystal structures with small molecules have been reported 88. USP7 has also been implicated in the regulation of anti-tumor immune responses. In this context, USP7 promotes the function of regulatory T cells, regulates polarization of tumor-associated macrophages, and the expression of programmed death-ligand 1 (PD-L1) in tumor cells 89.

#### USP8

USP8 removes K48 and K63 linked poly-Ub chains from substrates thereby participating in refilling of the ubiquitin cell pool and has been implicated in EGFR regulation and endosomal sorting and in protection from degradation 13, 14. Besides, it has been demonstrated that USP8 is phosphorylated in Akt-dependent manner following neuregulin stimulation 15. Of note, USP8 is overexpressed in various cancer types and in ovarian cancer it is associated with poor prognosis 90. Targeting of USP8 reduces survival pathway activation in ovarian carcinoma preclinical models of cisplatin resistance 90, offering novel opportunities for drug development in this disease and in many neoplastic diseases with a key role for EGFR activation including for example lung cancer in which down-regulation of the HER family receptor phosphorylation was observed following molecular and pharmacological inhibition of USP8 91. Although USP8 targeting may appear not amenable due to the essential role of this gene in cell viability as shown by embryonic lethality in knockout mice 92, a certain degree of selectivity could be provided by increased expression in tumors. Thus, in specific context, this DUB seems to be a desirable anticancer target. Indeed, it has been implicated in the pathogenesis of corticotroph adenoma in which activating mutations sustaining the EGFR pathways stimulation are reported 93. USP8 has been also implicated in the regulation of apoptotic cell death 94 that can results from decreased survival signals by receptor tyrosine kinases, but also from changes in stability of anti-apoptotic proteins. Indeed, knockdown of USP8 in ovarian cancer cells produces a downregulation of the anti-apoptotic action of FLIP_L_ associated with enhanced susceptibility to cisplatin-induced apoptosis resulting from enhanced caspase 3/7 activation 90. In this regard, USP8 has been reported to counteract apoptosis mediated by death receptors by increasing the stability of FLIP_S_ 95 and this is consistent with enhanced cisplatin-induced activation of caspase 8 following USP8 knockdown in ovarian carcinoma cells 90. In addition, a modulation of the efficacy of immunotherapy can be foreseen because USP8 inhibition has been recently reported to reshape an inflamed tumor microenvironment 96. Indeed, USP8 negatively regulates PD-L1 by removing K63-linked Ub resulting in increased K48-linked ubiquitin and PD-L1 degradation. Besides, pharmacological or molecular inhibition enhances PD-L1 but also increases innate and adaptive immune responses 96.

#### USP28

USP28 has been recently identified as a down-stream target gene of the β-catenin-YAP1-TBX5 transcriptional complex 97. USP28 expression is increased in ovarian cancer as compared to normal tissue and enhanced expression has been associated with a poor prognosis 97. Deregulated USP28 expression is linked to the activation of β-catenin signaling pathway. In fact, the expression of USP28 has been highly correlated with the CTNNB1 gene that codes for β-catenin as well as CCND1 and BIRC5, both targets of the Wnt/β-catenin signaling pathway. In addition, it was hypothesized and then confirmed that USP28 was a target gene for the transcription factor TBX5 as USP28 promoter indeed contains binding sites for TBX5.

#### USP35

An additional USP that has revealed promising features as a therapeutic target in ovarian carcinoma is USP35. High levels of USP35 have been reported to correlate with reduced infiltration of CD8^+^ T cells and poor survival 98 in tumor specimens. Analysis of public databases indicates that USP35 is frequently amplified in ovarian cancer and expressed at higher levels than in normal cells, but increased levels can be found in other cancer types such as renal cell carcinoma and gastric cancer 99, 100. In renal carcinoma, USP35 has shown the capability to stabilize multiple members of the IAP family, so that its loss results in enhanced apoptosis 99. In gastric cancer, USP35 has been implicated in Snail stabilization that appears to favor metastatic spread. 100. In ovarian cancer, the interferon (IFN) pathway has been found to be enriched in samples with low USP35, suggesting a role of USP35 in preventing recruitment of immune cells 98. Indeed, functional studies using knockout or over-expressing cells indicates a role for USP35 in negatively regulating IFN signaling. Of note, USP35 was found to deubiquitinate stimulator of interferon genes (STING) after direct binding and to regulate the STING-TBK-IRF3 pathways as their phosphorylation was enhanced upon USP35 knockdown in response to cGAMP, but abolished upon USP35 over-expression. Moreover, USP35 was found to modulate STING-mediated interferon with IFN-β and Cxcl10 levels increased in tumors from mice inoculated with USP35 knockdown cells.

#### USP39

USP39 is a DUB involved in spliceosomal complex assembly and characterized by the lack of catalytic activity due to the absence of catalytically-active amino acid residues; it is therefore one of the pseudoenzymes of the DUB family. It is known as a serine/arginine (SR) related protein because of the arginine, serine and glutamic acid rich N-terminal domain, similarly to SR proteins that participate in the recruitment of tri-snRNP to the pre-spliceosome 101. USP39 has been shown to be over-expressed in multiple tumor types 102, 103, including ovarian cancer, where it has been reported to be highly expressed in clinical specimens from patients resistant to carboplatin 104. In addition, USP39 has been reported to be increased in ovarian cancer as compared to normal tissue, with a positive relationship between levels and TNM stage, implying a possible role in disease prognosis 105. USP39 has been shown to be transcriptionally activated by c-Myc; its deletion results in impaired splicing as shown by skipped exons and intron and intergenic region over-representation 106. An oncogenic role for USP39 is supported by the evidence that it facilities the splicing of high mobility group A2 (HMGA2), an oncogenic co-activator of transcription 107. Functional approaches have demonstrated that USP39 plays a key role in supporting tumor cell proliferation and survival as demonstrated by short-hairpin mediated down regulation and over-expression using cell growth and colony forming assays. Indeed, knockdown of USP39 results in cell-cycle perturbation with G2/M arrest associated with increased cyclin B1 levels as well as increased platinum agent induced cell death linked to PARP and caspase 3 cleavage 104. Conversely, the over-expression of USP39 produces decreased apoptosis as well as enhanced activation of cell survival pathway mediated by EGFR activation as enhanced EGFR, Akt and ERK phosphorylation are observed. The available literature also suggests that USP39 promotes the progression of ovarian carcinoma cells via inhibition of the p53-p21 pathway and Wnt pathway with evidence of an impact of knockdown on the *in vivo* growth 105. Of note, the suppression of USP39 impairs the migratory cell abilities with inhibition of slug and E-cadherin leading to EMT inhibition.

#### USP48

Inhibition of migration of ovarian cancer cells following knockdown is also observed upon interference with USP48, another USP that has been investigated in this disease 108. In *in vitro* studies, it has been established that USP48 is implicated in the regulation of migration and invasion, but not of proliferation. However, knockdown of USP48 results in increased sensitivity to carboplatin, a feature associated with enhanced PARP and caspase-3 cleavage. Consistently, high USP48 levels appear to be related with resistance to carboplatin. Differently from what is observed for other DUBs, the over-expression of USP48 does not result in increased migration or resistance, suggesting a sort of saturation state in terms of expression. Besides, high USP48 levels are associated with poor prognosis both as progression-free and overall survival, in keeping with the observation from animal studies of reduced metastasis upon USP48 ablation. Thus, USP48 may represent a prognostic biomarker and may be exploitable for therapeutic targeting. The therapeutic relevance of USP48 has been also highlighted in FA, a rare disease with increased cancer risk caused by defective repair of DNA interstrand-crosslinks 109. In fact, USP48 is synthetic viable for FA-gene deficiencies and FA-deficient cells additionally lacking USP48 are less sensitive to cross-links induced genotoxic stress than FA-defective cells, showing increased clearance of DNA damage dependent on BRCA1.

### DUB targeting by small molecules

The design of inhibitors of DUBs is challenging considering several issues including the need to develop compounds selective for specific DUBs belonging to families of related enzymes 110. The catalytic site contains a reactive thiol group that may undergo redox reaction providing false positives in screenings 111. DUB enzymatic activity is regulated in a complex manner through allosteric and substrate-mediated effects 112.

**Figure 4:**
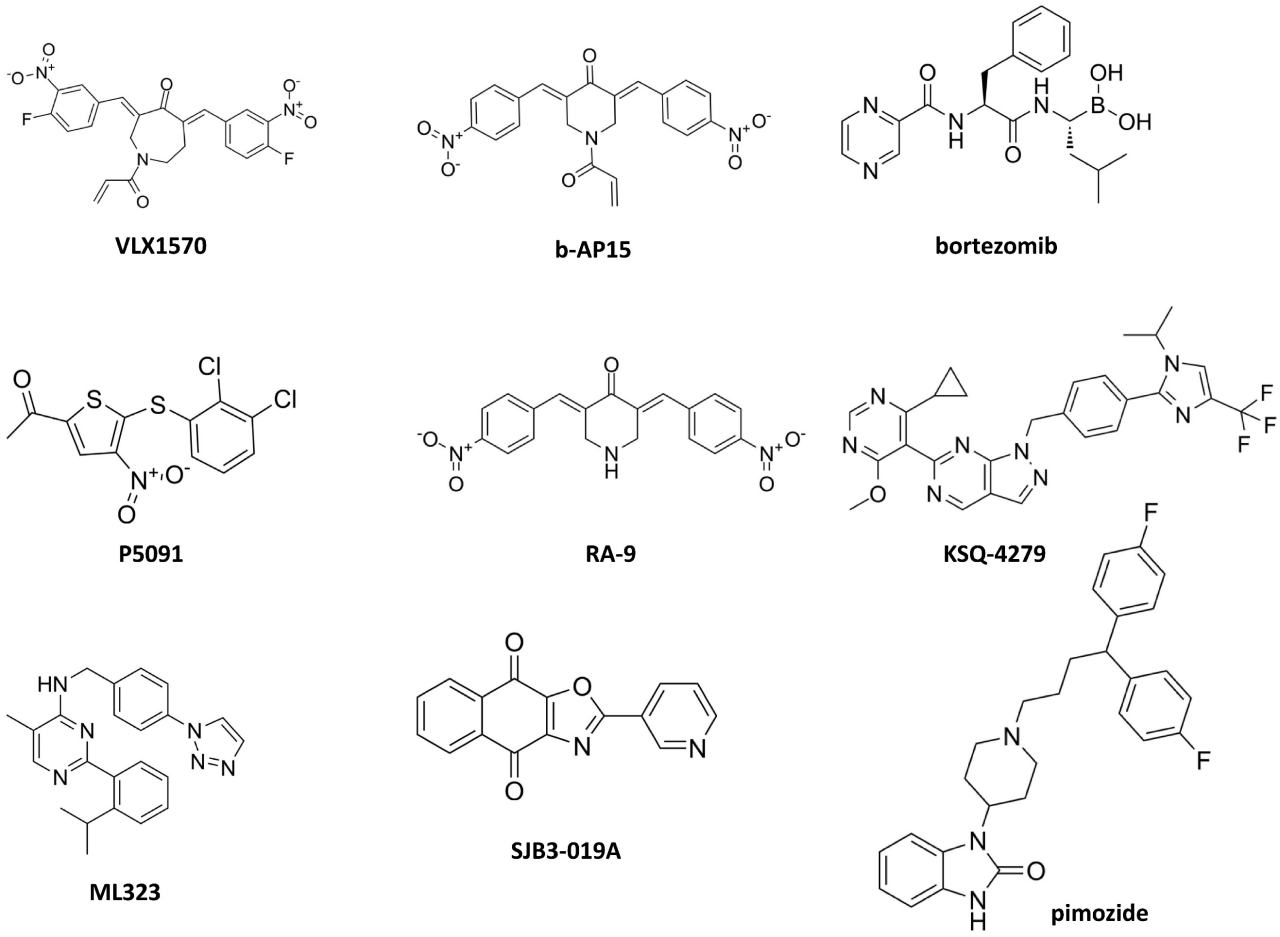
** Small molecules targeting deubiquitinases.** The chemical structures of compounds that have been investigated in ovarian cancer are reported. USP14 and UCHL5 inhibitors: VLX1570, b-AP15; 20S CP inhibitor: bortezomib; USP7 inhibitor: P5091; USP8 inhibitor: RA-9; USP1 inhibitors: KSQ-4279, ML323, SJB3-019A, pimozide.

The first DUB inhibitor that achieved clinical development is VLX1570 (Figure 4 and Table 2), a derivative of b-AP15 that inhibits both USP14 and UCHL5. The latter DUB has been associated with poor survival in ovarian cancer 113. In ovarian cancer preclinical models, b-AP15 has shown capability to inhibit survival of TP53 mutant cells by regulation of TGF-β signaling as a consequence of UCHL5 blockade and Smad2 phosphorylation resulting in apoptosis. Unfortunately, the clinical development of VLX1570 has been terminated for dose limiting toxicities 114, likely resulting from protein cross-linking 115, but the preclinical evidence of efficacy in different cancer types suggest the need to develop more tolerable inhibitors.

A recent review of patents of USP7 inhibitors highlights the molecular heterogeneity of the main scaffolds of these inhibitors that have been described also in natural products 88. First generation inhibitors have been reported (e.g., P5091) capable of overcoming resistance to proteasome inhibitors targeting the 20S CP (i.e., bortezomib) 116.

Second generation inhibitors have been already produced, and they are characterized by higher potency and selectivity as well as improved pharmacokinetics properties as compared to the first-generation ones. However, the available compounds which include reversible and irreversible inhibitors have not entered clinical development. Although the preclinical efficacy of small-molecule USP7 inhibitors was demonstrated *in vivo* and the synergistic effect of combining USP7 inhibition with cancer immunotherapy appears promising, the field seems to be at its infancy.

Inhibitors of other DUBs have been reported in the last decades including USP1, USP2, USP8, USP30, CSN5, STAMBP and RPN11/PSMD14 as well as multi-targeted USP inhibitors 117, 118. Coughlin and colleagues reported that the USP8 inhibitor RA-9 selectively promotes apoptosis in ovarian cancer cell lines and primary culture cells, and that its mechanism of action is related to an unfolded protein response activated against a proteotoxic stress. Although the *in vivo* results were promising, RA-9 did not reach the clinical development 118.

Efforts have been made on pharmacological targeting of USP1 119-126 and they may pave the way to new opportunities for ovarian cancer treatment. The development of a first in class USP1 inhibitor, i.e., KSQ-4279, capable to inhibit tumor growth in models partially sensitive to PARP inhibitors and to induce durable tumor regression in combination with PARP inhibitors is ongoing 119. Indeed, in a CRISPR screen employing over 700 tumor cell lines including a subgroup with BRCA1/2 mutation and DNA repair defects, USP1 was found as a suitable target based on genetic dependency. An acceptable safety profile for KSQ-4279 has been already reported 120. Given the preclinical evidence of activity of this inhibitor in tumors with HR deficiency, a contribution of such a type of inhibitors to the enrichment of the drugs available for ovarian carcinoma, particularly for high grade serous ovarian cancer that exhibits a high frequency of mutation (i.e., 50%) of HR genes, is expected. ML323 is a USP1 inhibitor that showed interesting antiproliferative activity on ovarian cancer cells by blocking S-phase cell-cycle 121. Recently, the analysis of the co-crystal structure of ML323 bound to USP1 122 has been instrumental to the characterization of the binding mode of KSQ-4279 and for the synthesis of novel pyrido[2,3-d]pyrimidin-7)8H)-one derivatives 123.

A recent paper has reported an association between sensitivity to USP1 inhibitors and the accumulation of single-strand DNA (ssDNA) gaps during replication in BRCA1 deficient cells; suppression of the gaps upon USP1 inhibition occurs following deubiquitination of PCNA 124. The mechanism of USP1 inhibitor-induced killing involved RAD18, an E3 ligase for PCNA ubiquitination that upon USP1 inhibition is trapped at replication forks and is likely to block the refilling of ssDNA gaps mediated by translesion synthesis polymerase. Thus, ssDNA gap accumulation may be exploited as a biomarker of response to USP1 inhibitors.

The discovery of new chemical entities capable of inhibiting DUBs is being pursued through innovative strategies that go beyond target-based screen, instead proposing target-class approach to DUB inhibitor development 125, 126. A chemoproteomic platform to test cysteine-reactive covalent fragments against DUBs using cell lysates through activity-based protein profiling has been recently developed 126. Targeted identification and covalent fragment library profiling carried out taking advantage of targeted ubiquitin probe allowed profiling of more than 100 covalent fragments *versus* 57 DUBs. The identified and validated hit fragments target OTUD7B and UCHL3. Pharmacological inhibitors of USP1 such as SJB3-019 and pimozide have been reported to reduce cisplatin-induced Snail expression in ovarian carcinoma cells similarly to what is observed upon USP1 knockdown 80.

## Conclusion and perspectives

Advances in the molecular characterization of tumors have expanded the knowledge about multiple mechanisms including ubiquitination and deubiquitination. In this context, a role for DUBs in specific tumor types such as ovarian cancer is clearly emerging. Several components of this large family of enzymes have already been reported to contribute to ovarian cancer aggressiveness and drug resistance. The most promising enzyme appearing in this scenario is USP1 that is upregulated in BRCA1/2 deficient tumors 127. In the absence of USP1 mono Ub PCNA at the replication fork causes cell death, whereas when present USP1 protects the fork and promotes cell survival. In keeping with this evidence, USP1 plays a pro-survival role dependent on EMT and stem cell state 80 as well as on the interaction with the microtubule-associated serine/threonine kinase 1 (MAST1) that extends MAST1 half-life by preventing K48-linked polyubiquitination and promotes MEK1 activation 128. The understanding of the biological relevance of USP1 in tumor cells has supported the preclinical development of various small molecules that are getting close to the clinical phase of development 129, 130. Although the development of USP14 inhibitors has been delayed by the termination of a trial with the VLX1570 compound, targeting of USP14 appears really promising also considering the preclinical evidence that it is not easy to develop resistance to this class of compounds and, eventually, low level of resistance can occur 131. Natural compounds or their derivatives may also display capability to interfere with DUBs as proved for the degradation product of curcumin, caffeic acid phenethyl ester that has been reported to inhibit USP8 132.

Several DUBs have been reported to contribute to the aggressiveness of ovarian cancer, a phenomenon that suggests that inhibitors capable of targeting multiple enzymes may be particularly promising. For some DUBs a clear role in conferring resistance to platinum agents has been recognized, and selective inhibitors could already have a major impact in shaping therapeutic strategies. However, biologically relevant results are already available for selected DUBs also from tumor types different from ovarian cancer. For instance, USP15 has been investigated in breast cancer where it has been reported to sustain oncogenesis and tumor growth by different mechanisms such as activation of TGF-β 133, stabilization of MDM2 134 and promotion of an immunosuppressive tumor microenvironment 135. Of note, USP15 appears to be a key regulator of HR repair because by deubiquitinating its major binding partner BARD1 promotes BRCA1/BARD1 retention at double-strand breaks facilitating double-strand break end resection, thereby allowing DNA repair 136.

Although DUBs have initially been explored as potential targets expressed by tumor cells, their contribution to main homeostatic mechanisms including immunological processes has gradually emerged. Indeed, besides the key role of USP8 in T cell development and homeostasis 16, USP15 levels have been shown to have an impact on antitumor cells responses, its deficiency being associated with excessive production of IFN-γ, which promotes an immunosuppressive tumor microenvironment in an experimental model of tumorigenesis 137. In line with a favorable role of USP15 at the tumor site, USP15 low expression was found to be linked to reduced CD8^+^ T cell infiltration and poor prognosis in triple negative breast cancer patients as a consequence of USP15 ability to inhibit PD-L1 transcription by deubiquitinating the transcriptional co-activator VGLL4, leading to increased CD8^+^ T cell infiltration resulting in increased efficacy of immunotherapy 138

A contribution of DUBs in regulating the stability of molecules expressed by tumors to evade immune surveillance is also possible. In this regard, USP22 appears to directly regulate PD-L1 levels by deubiquitination 139.

Overall, DUBs appear to be able to orchestrate different functions both at the tumor and microenvironment level acting both in adaptive and innate immunity mechanisms 98. The role for DUBs in the tumor microenvironment is also supported by the expression of USP7 by bone marrow metastatic melanoma cells 140. The development of inhibitors of this class of enzymes seems therefore to be extremely promising and to open strategies to simultaneously target the tumor and its microenvironment. Medicinal chemistry efforts in the field will provide also therapeutic opportunities in other diseases because DUBs have been shown to contribute to the pathogenesis of other diseases such as neurodegeneration 141.

## Figures and Tables

**Table 1:** Deubiquitinases and drug resistance

Cellular mechanism	Deubiquitinase	Role in drug resistance	Reference
Proteasomal deubiquitinases	USP14	●Regulation of cell proliferation and apoptosis. ●Stabilization of the oncoprotein Bcl6. ●Regulation of heme metabolism and of invasive cell ability via stabilization of BACH1 and activation of Nrf2.	68, 70, 73
	RPN11/PSMD14	●Inhibition of autophagy and sustaining of EMT through activation of LRPPRC/Beclin1-Bcl-2/SQSTM1 signaling pathway mediated by HAPSTR1. ●PKM2-mediated stimulation of the Warburg effect and enzyme nuclear translocation.	76, 77, 78
Non-proteasomal deubiquitinases	USP1	●Regulation of DDR by deubiquitinating PCNA and FANCD2. ●Regulation of EMT and stem cell state via stabilization of MAP3K7 and Snail.	81, 82, 83
	USP7	●Regulation of the p53/MDM2 axis. ●Regulation of T cell functions, polarization of tumor-associated macrophages as well as the expression of PD-L1 in tumor cells.	85, 89
	USP8	●Regulation of cell proliferation via EGFR activation. ●Regulation of apoptotic cell death via FLIPL, caspase 3/7 and 8. ●Generation of an inflamed tumor microenvironment.	90, 94, 96
	USP28	●Activation of β-catenin signalling pathway.	97
	USP35	●Reduced infiltration of immune cells by regulating STING-TBK-IRF3 pathway. ●Stabilization of multiple members of the IAP family. ●Stabilization of Snail favouring metastatic spread.	98, 99, 100
	USP39	●Promotion of transcription mediated by the splicing of HMGA2. ●Promotion of tumor cell proliferation and survival mediated by EGFR, Akt and ERK activation and resistance to apoptosis induction by the inhibition of the p53-p21 and Wnt pathways. ●Increased cell migration ability via slug and E-cadherin.	105, 107
	USP48	●Regulation of cell migration and invasion as well as resistance to apoptosis induction.	108

**Table 2:** Small molecules targeting deubiquitinases in ovarian cancer.

Target	Compound	Mechanism of action	Reference
USP14/UCHL5	VLX1570 b-AP15	Inhibition of survival of TP53 mutant cells by regulating TGF-β signaling and Smad2 phosphorylation leading to apoptosis.	113, 114, 115
USP7	P5091	Overcome resistance to 20 S CP inhibitors (e.g., bortezomib).	116
USP8	RA-9	Induction of apoptosis mediated by an unfolded protein response activated against a proteotoxic stress.	118
USP1	KSQ-4279	Inhibition of growth of tumor models partially sensitive to PARP inhibitors and induction of a durable tumor regression in combination with a PARP inhibitors.	119
	ML323	Induction of apoptosis via a S-phase cell-cycle block.	121
	SJB3-019A Pimozide	Reduction of cisplatin-induced Snail expression	80

## Abbreviations

DUBs: deubiquitinases
EMT: epithelial mesenchymal transition
PARP: Poly-ADP ribose polymerase
CAFs: cancer-associated fibroblasts
HR: homologous recombination
USP: Ubiquitin-Specific Protease
CP: cysteine proteases
MP: metallo proteases
UCH: ubiquitin C-terminal hydrolase
OTU: ovarian tumor domain-containing proteases
MJD: Machado-Josephin domain-containing proteases
MINDY: K48 polyubiquitin-specific MIU-containing the novel DUB family domain-containing proteases
UFM1: Ubiquitin Fold Modifier1-specific peptidase domain-containing proteases
Poly-Ub: polyubiquitin
ATXN3: ataxin 3
JAMM: zinc-dependent enzymes equipped with JAB1/MPN/MOV34
RPN11: regulatory particle non-ATPase 11
ESCRT: Endosomal Sorting Complexes Required for Transport
LRPRC: Leucine Rich Pentatricopeptide Repeat Containing
HAPSTR1: C16orf72/HUWE1-associated protein modifying stress responses
DDR: DNA damage response
TAK1: TGF-β-activated kinase 1
MAP3K7: mitogen-activated protein kinase 7
HAUSP: Herpesvirus associated Ubiquitin-Specific Protease
PD-L1: programmed death-ligand 1
IFN: interferon
STING: stimulator of interferon genes
SR: serine/arginine
HMGA2: high mobility group A2
FA: Fanconi Anemia
FANCD2: Fanconi anemia complementation group D2
MAST1: microtubule-associated serine/threonine kinase 1
ssDNA: single-strand DNA

## References

[1] Kroeger PT Jr, Drapkin R (2017). Pathogenesis and heterogeneity of ovarian cancer. Curr Opin Obstet Gynecol.

[2] Havasi A, Cainap SS, Havasi AT, Cainap C (2023). Ovarian Cancer-Insights into Platinum Resistance and Overcoming It. Medicina (Kaunas).

[3] Kurnit KC, Coleman RL, Westin SN (2018). Using PARP Inhibitors in the Treatment of Patients With Ovarian Cancer. Curr Treat Options Oncol.

[4] Rottenberg S, Disler C, Perego P (2021). The rediscovery of platinum-based cancer therapy. Nat Rev Cancer.

[5] Perego P, Giarola M, Righetti SC, Supino R, Caserini C, Delia D, Pierotti MA, Miyashita T, Reed JC, Zunino F (1996). Association between cisplatin resistance and mutation of p53 gene and reduced bax expression in ovarian carcinoma cell systems. Cancer Res.

[6] Scurry J, van Zyl B, Gulliver D, Otton G, Jaaback K, Lombard J, Vilain RE, Bowden NA (2018). Nucleotide excision repair protein ERCC1 and tumour-infiltrating lymphocytes are potential biomarkers of neoadjuvant platinum resistance in high grade serous ovarian cancer. Gynecol Oncol.

[7] Cossa G, Lanzi C, Cassinelli G, Carenini N, Arrighetti N, Gatti L, Corna E, Zunino F, Zaffaroni N, Perego P (2014). Differential outcome of MEK1/2 inhibitor-platinum combinations in platinum-sensitive and -resistant ovarian carcinoma cells. Cancer Lett.

[8] Konstantinopoulos PA, Matulonis UA (2023). Clinical and translational advances in ovarian cancer therapy. Nat Cancer.

[9] Jiang Y, Wang C, Zhou S (2020). Targeting tumor microenvironment in ovarian cancer: Premise and promise. Biochim Biophys Acta Rev Cancer.

[10] Wang W, Kryczek I, Dostál L, Lin H, Tan L, Zhao L, Lu F, Wei S, Maj T, Peng D, He G, Vatan L, Szeliga W, Kuick R, Kotarski J, Tarkowski R, Dou Y, Rattan R, Munkarah A, Liu JR, Zou W (2016). Effector T Cells Abrogate Stroma-Mediated Chemoresistance in Ovarian Cancer. Cell.

[11] Dewson G, Eichhorn PJA, Komander D (2023). Deubiquitinases in cancer. Nat Rev Cancer.

[12] Hanahan D, Weinberg RA (2000). The hallmarks of cancer. Cell.

[13] Berlin I, Schwartz H, Nash PD (2010). Regulation of epidermal growth factor receptor ubiquitination and trafficking by the USP8•STAM complex. J Biol Chem.

[14] Row PE, Prior IA, McCullough J, Clague MJ, Urbé S (2006). The ubiquitin isopeptidase UBPY regulates endosomal ubiquitin dynamics and is essential for receptor down-regulation. J Biol Chem.

[15] Cao Z, Wu X, Yen L, Sweeney C, Carraway KL (2007). 3rd. Neuregulin-induced ErbB3 downregulation is mediated by a protein stability cascade involving the E3 ubiquitin ligase Nrdp1. Mol Cell Biol.

[16] Dufner A, Kisser A, Niendorf S, Basters A, Reissig S, Schönle A, Aichem A, Kurz T, Schlosser A, Yablonski D, Groettrup M, Buch T, Waisman A, Schamel WW, Prinz M, Knobeloch KP (2015). The ubiquitin-specific protease USP8 is critical for the development and homeostasis of T cells. Nat Immunol.

[17] Colombo N, Sessa C, du Bois A, Ledermann J, McCluggage WG, McNeish I, Morice P, Pignata S, Ray-Coquard I, Vergote I, Baert T, Belaroussi I, Dashora A, Olbrecht S, Planchamp F, Querleu D (2019). ESMO-ESGO Ovarian Cancer Consensus Conference Working Group. ESMO-ESGO consensus conference recommendations on ovarian cancer: pathology and molecular biology, early and advanced stages, borderline tumours and recurrent disease†. Ann Oncol.

[18] Ambroggio XI, Rees DC, Deshaies RJ (2004). JAMM: A metalloprotease-like zinc site in the proteasome and signalosome. PLoS Biol.

[19] Amerik AY, Hochstrasser M (2004). Mechanism and function of deubiquitinating enzymes. Biochim Biophys Acta.

[20] Johnston SC, Larsen CN, Cook WJ, Wilkinson KD, Hill CP (1997). Crystal structure of a deubiquitinating enzyme (human UCH-L3) at 1.8 A resolution. EMBO J.

[21] Pickart CM, Rose IA (1986). Mechanism of ubiquitin carboxyl-terminal hydrolase. Borohydride and hydroxylamine inactivate in the presence of ubiquitin. J Biol Chem.

[22] Schauer NJ, Magin RS, Liu X, Doherty LM, Buhrlage SJ (2020). Advances in discovering deubiquitinating enzyme (DUB) inhibitors. J Med Chem.

[23] Li Y, Reverter D (2021). Molecular mechanisms of DUBs regulation in signaling and disease. Int J Mol Sci.

[24] Suresh HG, Pascoe N, Andrews B (2020). The structure and function of deubiquitinases: Lessons from budding yeast. Open Biol.

[25] Ronau JA, Beckmann JF, Hochstrasser M (2016). Substrate specificity of the ubiquitin and Ubl proteases. Cell Res.

[26] Komander D, Clague MJ, Urbe S (2009). Breaking the chains: Structure and function of the deubiquitinases. Nat Rev Mol Cell Biol.

[27] Hu M, Li P, Li M, Li W, Yao T, Wu JW, Gu W, Cohen RE, Shi Y (2002). Crystal structure of a UBP-family deubiquitinating enzyme in isolation and in complex with ubiquitin aldehyde. Cell.

[28] Boudreaux DA, Maiti TK, Davies CW, Das C (2010). Ubiquitin vinyl methyl ester binding orients the misaligned active site of the ubiquitin hydrolase UCHL1 into productive conformation. Proc Natl Acad Sci USA.

[29] Maiti TK, Permaul M, Boudreaux DA, Mahanic C, Mauney S, Das C (2011). Crystal structure of the catalytic domain of UCHL5, a proteasome-associated human deubiquitinating enzyme, reveals an unproductive form of the enzyme. FEBS J.

[30] Nijman SM, Luna-Vargas MP, Velds A, Brummelkamp TR, Dirac AM, Sixma TK, Bernards R (2005). A genomic and functional inventory of deubiquitinating enzymes. Cell.

[31] Eletr ZM, Wilkinson KD (2014). Regulation of proteolysis by human deubiquitinating enzymes. Biochim Biophys Acta.

[32] Zhang W, Sulea T, Tao L, Cui Q, Purisima EO, Vongsamphanh R, Lachance P, Lytvyn V, Qi H, Li Y, Menard R (2011). Contribution of active site residues to substrate hydrolysis by USP2: Insights into catalysis by ubiquitin specific proteases. Biochemistry.

[33] Johnston SC, Riddle SM, Cohen RE, Hill CP (1999). Structural basis for the specificity of ubiquitin C-terminal hydrolases. EMBO J.

[34] Yu H, Mashtalir N, Daou S, Hammond-Martel I, Ross J, Sui G, Hart GW, Rauscher FJ 3rd, Drobetsky E, Milot E, Shi Y, Affar El B (2010). The ubiquitin carboxyl hydrolase BAP1 forms a ternary complex with YY1 and HCF-1 and is a critical regulator of gene expression. Mol Cell Biol.

[35] Masoomian B, Shields JA, Shields CL (2018). Overview of BAP1 cancer predisposition syndrome and the relationship to uveal melanoma. J Curr Ophthalmol.

[36] Mirra L, Beretta GL, Lisini D, Marcianti A, Spampinato E, Corno C, Costantino M, Corsico A, Stella GM, Perego P (2024). Therapeutic Strategies to Improve the Treatment of Pleural Mesothelioma. Curr Med Chem.

[37] Hu M, Li P, Song L, Jeffrey PD, Chenova TA, Wilkinson KD, Cohen RE, Shi Y (2005). Structure and mechanisms of the proteasome-associated deubiquitinating enzyme USP14. EMBO J.

[38] Renatus M, Parrado SG, D'Arcy A, Eidhoff U, Gerhartz B, Hassiepen U, Pierrat B, Riedl R, Vinzenz D, Worpenberg S, Kroemer M (2006). Structural basis of ubiquitin recognition by the deubiquitinating protease USP2. Structure.

[39] Makarova KS, Aravind L, Koonin EV (2000). A novel superfamily of predicted cysteine proteases from eukaryotes, viruses and Chlamydia pneumoniae. Trends Biochem Sci.

[40] Mevissen TE, Hospenthal MK, Geurink PP, Elliott PR, Akutsu M, Arnaudo N, Ekkebus R, Kulathu Y, Wauer T, El Oualid F, Freund SM, Ovaa H, Komander D (2013). OTU deubiquitinases reveal mechanisms of linkage specificity and enable ubiquitin chain restriction analysis. Cell.

[41] Edelmann MJ, Iphofer A, Akutsu M, Altun M, di Gleria K, Kramer HB, Fiebiger E, Dhe-Paganon S, Kessler BM (2009). Structural basis and specificity of human otubain 1-mediated deubiquitination. Biochem J.

[42] Bremm A, Freund SM, Komander D (2010). Lys11-linked ubiquitin chains adopt compact conformations and are preferentially hydrolyzed by the deubiquitinase Cezanne. Nat Struct Mol Biol.

[43] Nanao MH, Tcherniuk SO, Chroboczek J, Dideberg O, Dessen A, Balakirev MY (2004). Crystal structure of human otubain 2. EMBO Rep.

[44] Lin SC, Chung JY, Lamothe B, Rajashankar K, Lu M, Lo YC, Lam AY, Darnay BG, Wu H (2008). Molecular basis for the unique deubiquitinating activity of the NF-kappaB inhibitor A20. J Mol Biol.

[45] Albrecht M, Golatta M, Wullner U, Lengauer T (2004). Structural and functional analysis of ataxin-2 and ataxin-3. Eur J Biochem.

[46] Kawaguchi Y, Okamoto T, Taniwaki M, Aizawa M, Inoue M, Katayama S, Kawakami H, Nakamura S, Nishimura M, Akiguchi I, Kimura J, Narumiya S, Kakizuka A (1994). CAG expansions in a novel gene for Machado-Joseph disease at chromosome 14q32.1. Nat Genet.

[47] Maurer T, Wertz IE (2016). Length matters: MINDY is a new deubiquitinase family that preferentially cleaves long polyubiquitin chains. Mol Cell.

[48] Abdul Rehman SA, Kristariyanto YA, Choi SY, Nkosi PJ, Weidlich S, Labib K, Hofmann K, Kulathu Y (2016). MINDY-1 is a member of an evolutionarily conserved and structurally distinct new family of deubiquitinating enzymes. Mol Cell.

[49] Kwasna D, Abdul Rehman S.A, Natarajan J, Matthews S, Madden R, De Cesare V, Weidlich S, Virdee S, Ahel I, Gibbs-Seymour I, Kulathu Y (2018). Discovery and characterization of ZUFSP/ZUP1, a distinct deubiquitinase class important for genome stability. Mol Cell.

[50] Tran HJ, Allen MD, Lowe J, Bycroft M (2003). Structure of the Jab1/MPN domain and its implications for proteasome function. Biochemistry.

[51] Shrestha RK, Ronau JA, Davies CW, Guenette RG, Strieter ER, Paul LN, Das C (2014). Insights into the mechanism of deubiquitination by JAMM deubiquitinases from cocrystal structures of the enzyme with the substrate and product. Biochemistry.

[52] Davies CW, Paul LN, Kim MI, Das C (2011). Structural and thermodynamic comparison of the catalytic domain of AMSH and AMSH-LP: Nearly identical fold but different stability. J Mol Biol.

[53] Verma R, Aravind L, Oania R, McDonald WH, Yates JR 3rd, Koonin EV, Deshaies RJ (2002). Role of Rpn11 metalloprotease in deubiquitination and degradation by the 26S proteasome. Science.

[54] Lee BH, Lee MJ, Park S, Oh DC, Elsasser S, Chen PC, Gartner C, Dimova N, Hanna J, Gygi SP, Wilson SM, King RW, Finley D (2010). Enhancement of proteasome activity by a small-molecule inhibitor of USP14. Nature.

[55] Yao T, Song L, Xu W, DeMartino GN, Florens L, Swanson SK, Washburn MP, Conaway RC, Conaway JW, Cohen RE (2006). Proteasome recruitment and activation of the Uch37 deubiquitinating enzyme by Adrm1. Nat Cell Biol.

[56] Arendt CS, Hochstrasser M (1999). Eukaryotic 20S proteasome catalytic subunit propeptides prevent active site inactivation by N-terminal acetylation and promote particle assembly. EMBO J.

[57] Livneh I, Cohen-Kaplan V, Cohen-Rosenzweig C, Avni N, Ciechanover A (2016). The life cycle of the 26S proteasome: From birth, through regulation and function, and onto its death. Cell Res.

[58] Yao T, Cohen RE (2002). A cryptic protease couples deubiquitination and degradation by the proteasome. Nature.

[59] Worden EJ, Dong KC, Martin A (2017). An AAA motor-driven mechanical switch in Rpn11 controls deubiquitination at the 26S proteasome. Mol Cell.

[60] Finley D (2009). Recognition and processing of ubiquitin-protein conjugates by the proteasome. Annu Rev Biochem.

[61] Lee MJ, Lee BH, Hanna J, King RW, Finley D (2011). Trimming of ubiquitin chains by proteasome-associated deubiquitinating enzymes. Mol Cell Proteomics.

[62] Wang L, Chen YJ, Xu K, Wang YY, Shen XZ, Tu RQ (2014). High expression of UCH37 is significantly associated with poor prognosis in human epithelial ovarian cancer. Tumour Biol.

[63] McCullough J, Clague MJ, Urbe S (2004). AMSH is an endosome-associated ubiquitin isopeptidase. J Cell Biol.

[64] McCullough J, Row PE, Lorenzo O, Doherty M, Beynon R, Clague MJ, Urbe S (2006). Activation of the endosome-associated ubiquitin isopeptidase AMSH by STAM, a component of the multivesicular body-sorting machinery. Curr Biol.

[65] McDonell LM, Mirzaa GM, Alcantara D, Schwartzentruber J, Carter MT, Lee LJ, Clericuzio CL, Graham JM Jr, Morris-Rosendahl DJ, Polster T, Acsadi G, Townshend S, Williams S, Halbert A, Isidor B (2013). Mutations in STAMBP, encoding a deubiquitinating enzyme, cause microcephaly-capillary malformation syndrome. Nat Genet.

[66] D'Arcy P, Brnjic S, Olofsson MH, Fryknäs M, Lindsten K, De Cesare M, Perego P, Sadeghi B, Hassan M, Larsson R, Linder S (2011). Inhibition of proteasome deubiquitinating activity as a new cancer therapy. Nat Med.

[67] Yang Y, Hou JQ, Qu LY, Wang GQ, Ju HW, Zhao ZW, Yu ZH, Yang HJ (2007). (Differential expression of USP2, USP14 and UBE4A between ovarian serous cystadenocarcinoma and adjacent normal tissues). Xi Bao Yu Fen Zi Mian Yi Xue Za Zhi.

[68] Wang Y, Wang J, Zhong J, Deng Y, Xi Q, He S, Yang S, Jiang L, Huang M, Tang C, Liu R (2015). Ubiquitin-specific protease 14 (USP14) regulates cellular proliferation and apoptosis in epithelial ovarian cancer. Med Oncol.

[69] Luo H, Wang X, Ge H, Zheng N, Peng F, Fu Y, Tao L, Wang Q (2019). Inhibition of ubiquitin-specific protease 14 promotes connexin 32 internalization and counteracts cisplatin cytotoxicity in human ovarian cancer cells. Oncol Rep.

[70] Shen J, Hong L, Chen L (2020). Ubiquitin-specific protease 14 regulates ovarian cancer cisplatin-resistance by stabilizing BCL6 oncoprotein. Biochem Biophys Res Commun.

[71] Wang YQ, Xu MD, Weng WW, Wei P, Yang YS, Du X (2014). BCL6 is a negative prognostic factor and exhibits pro-oncogenic activity in ovarian cancer. Am J Cancer Res.

[72] Wu M, Xie J, Xing Y, Zhang L, Chen H, Tang B, Zhou M, Lv S, Huang D, Jian S, Zhou C, Liu M, Guo W, Chen Y, Yi Z (2024). Selectively targeting BCL6 using a small molecule inhibitor is a potential therapeutic strategy for ovarian cancer. Int J Biol Sci.

[73] Ji J, Lv J, Lv M, Jing A, Xu M, Yuan Q, Ma X, Qian Q, Wang W, Geng T, Ding Y, Qin J, Liu Y, Yang J, Zhou J, Ma L, Wang Y, Zuo L, Wang X, Ma S, Liu B (2023). USP14 regulates heme metabolism and ovarian cancer invasion through BACH1 deubiquitination and stabilization. Biochem Biophys Res Commun.

[74] D'Arcy P, Linder S (2012). Proteasome deubiquitinases as novel targets for cancer therapy. Int J Biochem Cell Biol.

[75] Spataro V, Simmen K, Realini CA (2002). The essential 26S proteasome subunit Rpn11 confers multidrug resistance to mammalian cells. Anticancer Res.

[76] Zhao Z, Xu H, Wei Y, Sun L, Song Y (2023). Deubiquitylase PSMD14 inhibits autophagy to promote ovarian cancer progression via stabilization of LRPPRC. Biochim Biophys Acta Mol Basis Dis.

[77] Li D, Wang M (2024). An LRPPRC-HAPSTR1-PSMD14 interaction regulates tumor progression in ovarian cancer. Aging (Albany NY).

[78] Sun T, Liu Z, Bi F, Yang Q (2021). Deubiquitinase PSMD14 promotes ovarian cancer progression by decreasing enzymatic activity of PKM2. Mol Oncol.

[79] Huang TT, Nijman SM, Mirchandani KD, Galardy PJ, Cohn MA, Haas W, Gygi SP, Ploegh HL, Bernards R, D'Andrea AD (2006). Regulation of monoubiquitinated PCNA by DUB autocleavage. Nat Cell Biol.

[80] Sonego M, Pellarin I, Costa A, Vinciguerra GLR, Coan M, Kraut A, D'Andrea S, Dall'Acqua A, Castillo-Tong DC, Califano D, Losito S, Spizzo R, Couté Y, Vecchione A, Belletti B, Schiappacassi M, Baldassarre G (2019). USP1 links platinum resistance to cancer cell dissemination by regulating Snail stability. Sci Adv.

[81] Liang F, Miller AS, Longerich S, Tang C, Maranon D, Williamson EA, Hromas R, Wiese C, Kupfer GM, Sung P (2019). DNA requirement in FANCD2 deubiquitination by USP1-UAF1-RAD51AP1 in the Fanconi anemia DNA damage response. Nat Commun.

[82] Han D, Wang L, Chen B, Zhao W, Liang Y, Li Y, Zhang H, Liu Y, Wang X, Chen T, Li C, Song X, Luo D, Li Z, Yang Q (2021). USP1-WDR48 deubiquitinase complex enhances TGF-β induced epithelial-mesenchymal transition of TNBC cells via stabilizing TAK1. Cell Cycle.

[83] Simoneau A, Engel JL, Bandi M, Lazarides K, Liu S, Meier SR, Choi AH, Zhang H, Shen B, Martires L, Gotur D, Pham TV, Li F, Gu L, Gong S, Zhang M, Wilker E, Pan X, Whittington DA, Throner S, Maxwell JP, Chen Y, Yu Y, Huang A, Andersen JN, Feng T (2023). Ubiquitinated PCNA Drives USP1 Synthetic Lethality in Cancer. Mol Cancer Ther.

[84] Saha G, Roy S, Basu M, Ghosh MK (2023). USP7 - a crucial regulator of cancer hallmarks. Biochim Biophys Acta Rev Cancer.

[85] Cummins JM, Vogelstein B (2004). HAUSP is required for p53 destabilization. Cell Cycle.

[86] Ma M, Yu N (2016). Ubiquitin-specific protease 7 expression is a prognostic factor in epithelial ovarian cancer and correlates with lymph node metastasis. Onco Targets Ther.

[87] Pozhidaeva A, Bezsonova I (2019). USP7: Structure, substrate specificity, and inhibition. DNA Repair (Amst).

[88] Li P, Liu Y, Liu HM (2022). A patent review of ubiquitin-specific protease 7 (USP7) inhibitors (2014-present). Expert Opin Ther Pat.

[89] Korenev G, Yakukhnov S, Druk A, Golovina A, Chasov V, Mirgayazova R, Ivanov R, Bulatov E (2022). USP7 Inhibitors in Cancer Immunotherapy: Current Status and Perspective. Cancers (Basel).

[90] Corno C, D'Arcy P, Bagnoli M, Paolini B, Costantino M, Carenini N, Corna E, Alberti P, Mezzanzanica D, Colombo D, Linder S, Arrighetti N, Perego P (2022). The deubiquitinase USP8 regulates ovarian cancer cell response to cisplatin by suppressing apoptosis. Front Cell Dev Biol.

[91] Byun S, Lee SY, Lee J, Jeong CH, Farrand L, Lim S, Reddy K, Kim JY, Lee MH, Lee HJ, Bode AM, Won Lee K, Dong Z (2013). USP8 is a novel target for overcoming gefitinib resistance in lung cancer. Clin Cancer Res.

[92] Dufner A, Knobeloch KP (2019). Ubiquitin-specific protease 8 (USP8/UBPy): a prototypic multidomain deubiquitinating enzyme with pleiotropic functions. Biochem Soc Trans.

[93] Jian F, Cao Y, Bian L, Sun Q (2015). USP8: a novel therapeutic target for Cushing's disease. Endocrine.

[94] Jeong M, Lee EW, Seong D, Seo J, Kim JH, Grootjans S, Kim SY, Vandenabeele P, Song J (2017). USP8 suppresses death receptor-mediated apoptosis by enhancing FLIPL stability. Oncogene.

[95] Panner A, Crane CA, Weng C, Feletti A, Fang S, Parsa AT, Pieper RO (2010). Ubiquitin-specific protease 8 links the PTEN-Akt-AIP4 pathway to the control of FLIPS stability and TRAIL sensitivity in glioblastoma multiforme. Cancer Res.

[96] Xiong W, Gao X, Zhang T, Jiang B, Hu MM, Bu X, Gao Y, Zhang LZ, Xiao BL, He C, Sun Y, Li H, Shi J, Xiao X, Xiang B, Xie C, Chen G, Zhang H, Wei W, Freeman GJ, Shu HB, Wang H, Zhang J (2022). USP8 inhibition reshapes an inflamed tumor microenvironment that potentiates the immunotherapy. Nat Commun.

[97] Shen J, Xie M, Xu Y, Qian Q, Qiu T, Shi W, Ren D, Ji J, Huang J (2023). Identification of the deubiquitinase USP28 as a novel molecular therapeutic target of ovarian cancer. Biochem Biophys Res Commun.

[98] Zhang J, Chen Y, Chen X, Zhang W, Zhao L, Weng L, Tian H, Wu Z, Tan X, Ge X, Wang P, Fang L (2021). Deubiquitinase USP35 restrains STING-mediated interferon signaling in ovarian cancer. Cell Death Differ.

[99] Wang S, Wang T, Zhang X, Cheng S, Chen C, Yang G, Wang F, Wang R, Zhang Q, Yang D, Zhang Y, Liu S, Qin H, Liu Q, Liu H (2023). The deubiquitylating enzyme USP35 restricts regulated cell death to promote survival of renal clear cell carcinoma. Cell Death Differ.

[100] Ma C, Tian Z, Wang D, Gao W, Qian L, Zang Y, Xu X, Jia J, Liu Z (2024). Ubiquitin-specific Protease 35 Promotes Gastric Cancer Metastasis by Increasing the Stability of Snail1. Int J Biol Sci.

[101] Lygerou Z, Christophides G, Séraphin B (1999). A novel genetic screen for snRNP assembly factors in yeast identifies a conserved protein, Sad1p, also required for pre-mRNA splicing. Mol Cell Biol.

[102] Huang Y, Pan XW, Li L, Chen L, Liu X, Lu JL, Zhu XM, Huang H, Yang QW, Ye JQ, Gan SS, Wang LH, Hong Y, Xu DF, Cui XG (2016). Overexpression of USP39 predicts poor prognosis and promotes tumorigenesis of prostate cancer via promoting EGFR mRNA maturation and transcription elongation. Oncotarget.

[103] Remitha NPSI, Wiguna IGWW, Sadvika IGAS, Sadeva IGKA, Supadmanaba IGP, Wihandani DM (2023). Clinicopathological and Prognostic Significance of Ubiquitin-Specific Protease 39 Overexpression in solid Cancers: A Meta-Analysis. Asian Pac J Cancer Prev.

[104] Wang L, Chen T, Li X, Yan W, Lou Y, Liu Z, Chen H, Cui Z (2019). USP39 promotes ovarian cancer malignant phenotypes and carboplatin chemoresistance. Int J Oncol.

[105] Yan C, Yuan J, Xu J, Zhang G, Li X, Zhang B, Hu T, Huang X, Mao Y, Song G (2019). Ubiquitin-specific peptidase 39 regulates the process of proliferation and migration of human ovarian cancer via p53/p21 pathway and EMT. Med Oncol.

[106] Wang S, Wang Z, Li J, Qin J, Song J, Li Y, Zhao L, Zhang X, Guo H, Shao C, Kong B, Liu Z (2021). Splicing factor USP39 promotes ovarian cancer malignancy through maintaining efficient splicing of oncogenic HMGA2. Cell Death Dis.

[107] Zhang S, Mo Q, Wang X (2019). Oncological role of HMGA2 (Review). Int J Oncol.

[108] Lei X, Li X, Chen H, Liu Z (2020). USP48 Sustains Chemoresistance and Metastasis in Ovarian Cancer. Curr Cancer Drug Targets.

[109] Velimezi G, Robinson-Garcia L, Muñoz-Martínez F, Wiegant WW, Ferreira da Silva J, Owusu M, Moder M, Wiedner M, Rosenthal SB, Fisch KM, Moffat J, Menche J, van Attikum H, Jackson SP, Loizou JI (2018). Map of synthetic rescue interactions for the Fanconi anemia DNA repair pathway identifies USP48. Nat Commun.

[110] Harrigan JA, Jacq X, Martin NM, Jackson SP (2018). Deubiquitylating enzymes and drug discovery: emerging opportunities. Nat Rev Drug Discov.

[111] Sahtoe DD, Sixma TK (2015). Layers of DUB regulation. Trends Biochem Sci.

[112] Mevissen TET, Komander D (2017). Mechanisms of Deubiquitinase Specificity and Regulation. Annu Rev Biochem.

[113] Fukui S, Nagasaka K, Miyagawa Y, Kikuchi-Koike R, Kawata Y, Kanda R, Ichinose T, Sugihara T, Hiraike H, Wada-Hiraike O, Sasajima Y, Ayabe T (2019). The proteasome deubiquitinase inhibitor bAP15 downregulates TGF-β/Smad signaling and induces apoptosis via UCHL5 inhibition in ovarian cancer. Oncotarget.

[114] Rowinsky EK, Paner A, Berdeja JG, Paba-Prada C, Venugopal P, Porkka K, Gullbo J, Linder S, Loskog A, Richardson PG, Landgren O (2020). Phase 1 study of the protein deubiquitinase inhibitor VLX1570 in patients with relapsed and/or refractory multiple myeloma. Invest New Drugs.

[115] Ward JA, Pinto-Fernandez A, Cornelissen L, Bonham S, Díaz-Sáez L, Riant O, Huber KVM, Kessler BM, Feron O, Tate EW (2020). Re-Evaluating the Mechanism of Action of α,β-Unsaturated Carbonyl DUB Inhibitors b-AP15 and VLX1570: A Paradigmatic Example of Unspecific Protein Cross-linking with Michael Acceptor Motif-Containing Drugs. J Med Chem.

[116] Chauhan D, Tian Z, Nicholson B, Kumar KG, Zhou B, Carrasco R, McDermott JL, Leach CA, Fulcinniti M, Kodrasov MP, Weinstock J, Kingsbury WD, Hideshima T, Shah PK, Minvielle S, Altun M, Kessler BM, Orlowski R, Richardson P, Munshi N, Anderson KC (2012). A small molecule inhibitor of ubiquitin-specific protease-7 induces apoptosis in multiple myeloma cells and overcomes bortezomib resistance. Cancer Cell.

[117] Chen S, Liu Y, Zhou H (2021). Advances in the Development Ubiquitin-Specific Peptidase (USP) Inhibitors. Int J Mol Sci.

[118] Coughlin K, Anchoori R, Iizuka Y, Meints J, MacNeill L, Vogel RI, Orlowski RZ, Lee MK, Roden RB, Bazzaro M (2014). Small-molecule RA-9 inhibits proteasome-associated DUBs and ovarian cancer *in vitro* and *in vivo* via exacerbating unfolded protein responses. Clin Cancer Res.

[119] Cadzow L, Brenneman J, Sullivan P, Liu H, Shenker S, McGuire M, Grasberger P, Sinkevicius K, Hafeez N, Histen G, Chipumuro E, Hixon J, Krall E, Cogan S, Wilt J, Schlabach M, Stegmeier F, Olaharski A, Wylie A (2020). Development of KSQ-4279 as a first-in-class USP1 inhibitor for the treatment of BRCA-deficient cancers. Eur J Cancer.

[120] Yap TA, Lakhani NJ, Patnaik A, Lee EK, Gutierrez M, Moore KN, Carneiro BA, Hays JL, Huang M, LoRusso P, Wylie A, Cadzow L, Goulet M, Tobin E, Krieter O, Schmid D, Blake SM, Dieterich M, Jamois C, Harris PM (2024). First-in-human phase I trial of the oral first-in-class ubiquitin specific peptidase 1 (USP1) inhibitor KSQ-4279 (KSQi), given as single agent (SA) and in combination with olaparib (OLA) or carboplatin (CARBO) in patients (pts) with advanced solid tumors, enriched for deleterious homologous recombination repair (HRR) mutations. DEVELOPMENTAL THERAPEUTICS—MOLECULARLY TARGETED AGENTS AND TUMOR BIOLOGY. https://doi.org/10.1200/JCO.

[121] Song B, Jiang Y, Jiang Y, Lin Y, Liu J (2022). ML323 suppresses the progression of ovarian cancer via regulating USP1-mediated cell cycle. Front Genet.

[122] Rennie ML, Arkinson C, Chaugule VK, Walden H (2022). Cryo-EM reveals a mechanism of USP1 inhibition through a cryptic binding site. Sci Adv.

[123] Li H, Liu BJ, Xu J, Song SS, Ba R, Zhang J, Huan XJ, Wang D, Miao ZH, Liu T, He JX, Xiong B (2024). Design, synthesis, and biological evaluation of pyrido[2,3-d]pyrimidin-7(8H)-one derivatives as potent USP1 inhibitors. Eur J Med Chem.

[124] da Costa AABA, Somuncu O, Ravindranathan R, Mukkavalli S, Martignetti DB, Nguyen H, Jiao Y, Lamarre BP, Sadatrezaei G, Moreau L, Liu J, Iyer DR, Lazaro JB, Shapiro GI, Parmar K, D'Andrea AD (2024). Single-stranded DNA Gap Accumulation is a Functional Biomarker for USP1 Inhibitor Sensitivity. Cancer Res.

[125] Chan WC, Liu X, Magin RS, Girardi NM, Ficarro SB, Hu W, Tarazona Guzman MI, Starnbach CA, Felix A, Adelmant G, Varca AC, Hu B, Bratt AS, DaSilva E, Schauer NJ, Jaen Maisonet I, Dolen EK, Ayala AX, Marto JA, Buhrlage SJ (2023). Accelerating inhibitor discovery for deubiquitinating enzymes. Nat Commun.

[126] Cookson R, Vuorinen A, Pettinger J, Kennedy CR, Kirkpatrick JM, Peltier-Heap RE, Powell A, Snijders AP, Skehel M, House D, Rittinger K, Bush JT (2023). A chemoproteomic platform for selective deubiquitinase inhibitor discovery. Cell Reports Physical Science 4. 2023; 101636. doi: org/10.1016/j. xcrp.

[127] Lim KS, Li H, Roberts EA, Gaudiano EF, Clairmont C, Sambel LA, Ponnienselvan K, Liu JC, Yang C, Kozono D, Parmar K, Yusufzai T, Zheng N, D'Andrea AD (2018). USP1 Is Required for Replication Fork Protection in BRCA1-Deficient Tumors. Mol Cell.

[128] Tyagi A, Kaushal K, Chandrasekaran AP, Sarodaya N, Das S, Park CH, Hong SH, Kim KS, Ramakrishna S (2022). CRISPR/Cas9-based genome-wide screening for deubiquitinase subfamily identifies USP1 regulating MAST1-driven cisplatin-resistance in cancer cells. Theranostics.

[129] Kan Z, Bai S, Ma K, Chen B, Jiang B, Wang F, Ma X, Zhou D, Shi C, Nie Y, Wang Y, Liu R, Sun J, Zhou H, Xu M, Li J, Liu B (2024). XZP-6924: A potent and highly selective USP1 inhibitor with the potential to improve efficacy and overcome resistance to PARP inhibitors Proceedings of the American Association for Cancer Research Annual Meeting 2024; Part 2; 2024 Apr 5-10; San Diego, CA. Philadelphia (PA): AACR; Cancer Res.

[130] Sun Q, Chen Y, Wu M, Lv J, Jin H, Zeng P, Song X, Hou W, Huang Q, Niu W, Zhang M, Wang TL, Chen A (2024). ASN-3186 is a potent and selective inhibitor of USP1 for the treatment of BRCA1/2 mut and HRD^+^ cancers. Proceedings of the American Association for Cancer Research Annual Meeting 2024; Part 1. 2024 Apr 5-10; San Diego, CA. Philadelphia (PA): AACR; Cancer Res.

[131] Mofers A, Perego P, Selvaraju K, Gatti L, Gullbo J, Linder S, D'Arcy P (2019). Analysis of determinants for *in vitro* resistance to the small molecule deubiquitinase inhibitor b-AP15. PLoS One.

[132] Colombo D, Gatti L, Sjöstrand L, Carenini N, Costantino M, Corna E, Arrighetti N, Zuccolo M, De Cesare M, Linder S, D'Arcy P, Perego P (2022). Caffeic acid phenethyl ester targets ubiquitin-specific protease 8 and synergizes with cisplatin in endometrioid ovarian carcinoma cells. Biochem Pharmacol.

[133] Eichhorn PJ, Rodón L, Gonzàlez-Juncà A, Dirac A, Gili M, Martínez-Sáez E, Aura C, Barba I, Peg V, Prat A, Cuartas I, Jimenez J, García-Dorado D, Sahuquillo J, Bernards R, Baselga J, Seoane J (2012). USP15 stabilizes TGF-β receptor I and promotes oncogenesis through the activation of TGF-β signaling in glioblastoma. Nat Med.

[134] Zou Q, Jin J, Hu H, Li HS, Romano S, Xiao Y, Nakaya M, Zhou X, Cheng X, Yang P, Lozano G, Zhu C, Watowich SS, Ullrich SE, Sun SC (2014). USP15 stabilizes MDM2 to mediate cancer-cell survival and inhibit antitumor T cell responses. Nat Immunol.

[135] Zou Q, Jin J, Xiao Y, Zhou X, Hu H, Cheng X, Kazimi N, Ullrich SE, Sun SC (2015). T Cell Intrinsic USP15 Deficiency Promotes Excessive IFN-gamma Production and an Immunosuppressive Tumor Microenvironment in MCA-Induced Fibrosarcoma. Cell Rep.

[136] Peng Y, Liao Q, Tan W, Peng C, Hu Z, Chen Y, Li Z, Li J, Zhen B, Zhu W, Li X, Yao Y, Song Q, Liu C, Qi X, He F, Pei H (2019). The deubiquitylating enzyme USP15 regulates homologous recombination repair and cancer cell response to PARP inhibitors. Nat Commun.

[137] Zou Q, Jin J, Xiao Y, Zhou X, Hu H, Cheng X, Kazimi N, Ullrich SE, Sun SC (2015). T Cell Intrinsic USP15 Deficiency Promotes Excessive IFN-γ Production and an Immunosuppressive Tumor Microenvironment in MCA-Induced Fibrosarcoma. Cell Rep.

[138] Wang X, Deng X, Hu J, Zheng W, Ye D, Zhou X, Fang L (2024). K48-linked deubiquitination of VGLL4 by USP15 enhances the efficacy of tumor immunotherapy in triple-negative breast cancer. Cancer Lett.

[139] Wang Y, Sun Q, Mu N, Sun X, Wang Y, Fan S, Su L, Liu X (2020). The deubiquitinase USP22 regulates PD-L1 degradation in human cancer cells. Cell Commun Signal.

[140] Vishnoi M, Boral D, Liu H, Sprouse ML, Yin W, Goswami-Sewell D, Tetzlaff MT, Davies MA, Oliva ICG, Marchetti D (2018). Targeting USP7 Identifies a Metastasis-Competent State within Bone Marrow-Resident Melanoma CTCs. Cancer Res.

[141] Bello AI, Goswami R, Brown SL, Costanzo K, Shores T, Allan S, Odah R, Mohan RD (2022). Deubiquitinases in Neurodegeneration. Cells.

